# Negotiating bodily sensations between patients and GPs in the context of standardized cancer patient pathways – an observational study in primary care

**DOI:** 10.1186/s12913-020-4893-4

**Published:** 2020-01-17

**Authors:** Cecilia Hultstrand, Anna-Britt Coe, Mikael Lilja, Senada Hajdarevic

**Affiliations:** 1 Department of Nursing, Umeå University, SE-901 87 Umeå, Sweden; 2 Department of Sociology, Umeå University, SE-901 87 Umeå, Sweden; 3 Department of Public Health and Clinical Medicine, Unit of Research, Education, and Development, Östersund Hospital, Umeå University, SE-901 87 Umeå, Sweden; 4 Department of Public Health and Clinical Medicine, Family Medicine, Umeå University, SE-901 87 Umeå, Sweden

**Keywords:** Interaction, Negotiation, Access, Primary healthcare, Cancer, Standardized care pathways

## Abstract

**Background:**

How interactions during patient-provider encounters in Swedish primary care construct access to further care is rarely explored. This is especially relevant nowadays since Standardized Cancer Patient Pathways have been implemented as an organizational tool for standardizing the diagnostic process and increase equity in access. Most patients with symptoms indicating serious illness as cancer initially start their diagnostic trajectory in primary care. Furthermore, cancer symptoms are diverse and puts high demands on general practitioners (GPs). Hence, we aim to explore how presentation of bodily sensations were constructed and legitimized in primary care encounters within the context of Standardized Cancer Patient Pathways (CPPs).

**Methods:**

Participant observations of patient-provider encounters (*n* = 18, on 18 unique patients and 13 GPs) were carried out at primary healthcare centres in one county in northern Sweden. Participants were consecutively sampled and inclusion criteria were i) patients (≥18 years) seeking care for sensations/symptoms that could indicate cancer, or had worries about cancer, Swedish speaking and with no cognitive disabilities, and ii) GPs who met with these patients during the encounter. A constructivist approach of grounded theory method guided the data collection and was used as a method for analysis, and the COREQ-checklist for qualitative studies (Equator guidelines) were employed.

**Results:**

One conceptual model emerged from the analysis, consisting of one core category Negotiating bodily sensations to legitimize access, and four categories i) Justifying care-seeking, ii) Transmitting credibility, iii) Seeking and giving recognition, and iv) Balancing expectations with needs. We interpret the four categories as social processes that the patient and GP constructed interactively using different strategies to negotiate. Combined, these four processes illuminate how access was legitimized by negotiating bodily sensations.

**Conclusions:**

Patients and GPs seem to be mutually dependent on each other and both patients’ expertise and GPs’ medical expertise need to be reconciled during the encounter. The four social processes reported in this study acknowledge the challenging task which both patients and primary healthcare face. Namely, negotiating sensations signaling possible cancer and further identifying and matching them with the best pathway for investigations corresponding as well to patients’ needs as to standardized routines as CPPs.

## Background

Patients with symptoms that can indicate cancer often seek care at primary healthcare centers (PHCs) as a first instance, making these the main gateway for timely diagnosis and access to secondary care. Most patients with diagnosed cancer present their sensations in primary care [1]. In Sweden, services that are available at PHCs include preventive actions and basic treatments as well as referral to further (secondary i.e. specialist) care [2].

Primary care practitioners consider cancer as a possible diagnosis almost every day in the clinical setting [1]. This is because cancer symptoms are diverse and are usually connected with “common” bodily sensations, for example, weight loss, diffuse pain, or feelings of not being well. Cancer diagnosis is a complex process that puts high demands on primary healthcare personnel [3, 4].

Adding to this complexity, in the Swedish context, Standardized Cancer Patient Pathways (CPPs) have been implemented since 2015 as an organizational tool for promoting early cancer diagnosis, increasing equity in cancer care, and endorsing patient satisfaction. The ambition with CPPs is to standardize the diagnostic process by regulating time frames for specified diagnostic procedures. Referral to secondary care through CPPs is based on well-defined symptoms, so called “alarm symptoms”, i.e. possible signs of cancer. CPPs intend to shorten the time interval between legitimate suspicion (presence of alarm symptoms) and start of treatment [3].

Lastly, primary care is often associated with GPs’ roles as gatekeepers. Gatekeeping can either restrict or permit referrals to secondary care by matching patients’ needs and demands with GPs’ assessment of what the following act will be. Gatekeeping functions by limiting access to specialist care, or conversely, building bridges between primary and secondary care [5, 6]. The implementation of CPPs may create new playing rules for general practitioners (GPs) by introducing a manual with which to identify people with symptoms indicating cancer (or when suspecting cancer) which might influence the encounter. Andersen and Vedsted [4] found that standardized guidelines (e.g. CPPs) combined with openness and being sensitive to patients’ presentation of experienced bodily sensations as prerequisites for improving timely diagnosis of cancer [4]. Thus, previous research illuminates the complexity of balancing gatekeeping, appropriate use of resources, person-centeredness, adherence to guidelines and patients’ wants.

### Interaction and negotiation during encounters

Bodily sensations are defined as a physiological experience, whereas symptoms are an interpretation and expression of these embodied sensations [7, 8]. The term symptoms refers to “subjective evidence of disease” (e.g. fatigue) while the term signs of illness refers to the “objective evidence of diseases” [8]. The presentation of experienced sensations is a crucial task for patients when interacting with GPs in primary care [8]. GPs meanwhile are tasked with interpreting the presented sensations and thereafter describing them for the patients from a medical perspective during the encounter [9]. Patients have expertise regarding their own bodies, and GPs can only gain access to these experiences through the patients’ presentation of them. GPs are thereby dependent on patients’ presentations to be able to make assessments [10, 11].

This turns attention to the ability of both patients and GPs to include each other and negotiate one another’s influence in their interactions during the encounter [12, 13]. Negotiation can therefore be understood as an ongoing process whereby individuals engage in interactions aimed to attain a certain outcome attainable only through the other party [14]. Negotiation further encompasses development of shared meanings, understandings and agreements, “getting things accomplished” based on joint interest and balance of power [15]. One goal of negotiating is to reach a mutual solution through the combination of expertise, power, understanding and compassion [14].

### Rationale

Previous research has mainly focused on individual behaviors of seeking care when suspecting cancer [16, 17], thereby overlooking factors that potentially could be of importance for further access to care, such as patient-provider interactions during encounters and standardized care pathways, such as CPPs. Additionally, standardized guidelines have been described as forcing healthcare professionals to fit patients’ health complaints into templates to legitimize access to further care [4, 18] and increasing access to further care for those patients who present alarm symptoms [19, 20]. This suggests that interaction and negotiation between patients and GPs regarding bodily sensations, during encounters in primary care influence the creation of access to further care. Nonetheless, we lack knowledge about how this occurs. By *creation of access to further care*, we are concerned not with determining the outcome of access (or lack thereof) but rather the processes underlying such outcomes.

### Aim

This study aimed to explore how presentation of bodily sensations were constructed and legitimized in primary care encounters within the context of Standardized Cancer Patient Pathways (CPPs).

## Methods

### Design

To reach our aim, this study employed observations in combination with a constructivist approach to grounded theory method (GTM) [21]. GTM with a constructivist approach assumes that both data and analysis are socially constructed and enable exploration of complex phenomena [22], such as negotiations and interactions between patient and GP. Observations are an appropriate method for capturing the process of presenting and legitimizing symptoms during encounters. An emergent design was employed, meaning that data was collected until saturation of our theoretical categories [21], and we followed the COREQ-checklist for qualitative studies according to Equator guidelines (See Additional file 1).

### Context and setting

Healthcare in Sweden is publicly available, tax funded and decentralized. Twenty-one regions share the responsibility of organizing healthcare. Primary care is most often the first instance for most care as well as the main gateway for accessing secondary care. There exist some private primary healthcare providers even though the majority of primary healthcare is publicly organized and financed. One of the main obstacles for Swedish primary care, especially in sparsely populated areas such as the northern region, is recruiting and maintaining personnel such as GPs.

Inhabitants in Sweden have the right to choose the PHC they want to be listed at, and as far as possible the PHC directs the patients all contacts and appointment to a personal doctor. To get an appointment in primary care the most common way is to call the PHC (that you have chosen to be listed at) and primary healthcare nurses will then schedule an appointment, if he or she assess it as necessary. Nowadays, patients can also log into the national web-based platform 1177.se, to make a request for an appointment in primary care, which is also managed and scheduled by a primary healthcare nurse.

Observations of patients’ first encounter at publicly available primary healthcare centers (PHCs) in one county in northern Sweden was carried out. Prior to the study, PHCs were initially invited to participate through an oral presentation of the study by the researchers during a visit to the PHCs. Written informed consent was thereafter obtained from the head of the PHC and from the GPs. In total, twelve PHC were recruited purposively to include PHCs located in both urban, semi-urban and rural areas. Six PHCs declined due to time constrains, six PHCs accepted to participate, and observations were conducted at four PHCs. Data collection was carried out between December 2017 and Mars 2019.

### Participants

Participants in this study, patients and GPs, were consecutively invited. We observed encounters with patients who sought care at the included PHCs for bodily sensation/symptom that could potentially indicate cancer or had worries about cancer, and GPs working at the included PHCs who met these patients during an encounter. Hence, inclusion criteria were i) patients (≥18 years) seeking care for sensations/symptoms that could indicate cancer, or had worries about cancer, Swedish speaking and with no cognitive disabilities, and ii) GPs who met with these patients.

### Procedure

Patients were initially briefly informed by the healthcare personnel about the study when the patients called the PHC to book an appointment, but after the booking had been made. Thereafter, the researchers met up with the patients at the PHCs in the waiting room before their booked appointment. Patients received oral and written information about the study and were given the opportunity to ask questions. Patients were informed that their decision around participating would not affect their given care. Written informed consent was then obtained from all participating patients. Participation was on voluntary basis, meaning that participants could withdraw from participating at any time. Twenty-six patients were asked to participate by having their encounter observed, of them 18 accepted. The GPs who were assigned an appointment with the participating patients were thus included in this study, in total 13 GPs participated. The GPs’ work experiences ranged between less than 1 to 27 years (median 4 years), and their age ranged between 26 and 62 years (median 38 years). See Table 1 for characteristics of the encounter and location of the PHCs. Ethical approval was granted from the regional ethical review board (Dnr. 2017–296-31 M/ 2018–242-32 M).

**Table 1:** Characteristics of the encounter; health complaint, sex of participants, and location of the PHCs

Reason for care-seeking (health complaint):	Urban	Semi-urban	Rural
Problems with stomach	♀ = 2		
Blood in stools	♂ = 1	♀ = 1	♀ = 1
Haematuria		♀ = 1	
Lump	♀ = 1	♀ = 1	♀ = 1 ♂ = 1
Weight loss	♀ = 1 ♂ = 1		
Skin lesion	♀ = 1 ♂ = 1		
Unusual tiredness	♀ = 2		
Coughing			♂ = 1
Constipation	♂ = 1		

Twelve women and six men participated at PHCs located at urban, semi-urban and rural areas

Observations were conducted by CH (PhD candidate) and SH (PI, Assoc. prof.), two researchers with different backgrounds (public health, health promotion and nursing). The observers had the role of “observer-as-participants” meaning that the observers were visible but passive [23] during the observations. CH had an outsider perspective (Etic), due to no experience of working within a clinical setting. SH had an insider perspective (Emic) due to a background as a registered nurse. In ethnographic field studies, the emic and the etic perspectives are considered as complementary to each other [23]. Four observations were, during the initial phase of data collection, conducted by both CH and SH, 13 were thereafter conducted solely by CH, and one conducted solely by SH.

In the beginning of the data collection process, CH and SH conducted descriptive and exploratory observations aiming to collect data in an as open manner as possible. Data was gathered on actions, communication (verbal and non-verbal) and other events that occurred during the encounter without doing any conscious decisions about what was noted.

Since it is essential to have a strategy to systematically collect data when conducting observations [23], we developed a protocol consisting of three sections. In the first section, actions related to the patients were noted. The second section was dedicated to GPs, and in the third section comments, analytical reflections, questions and thoughts from ourselves were written down. Instantly after the observations, we audio recorded our handwritten field notes from the protocol, which aided us to extend our written notes with our fresh memory of the encounter. These audits were then transcribed verbatim for analyzing the empirical material. Transcripts were then imputed to the software program MAXQDA version 2018 for coding, managing and analysis. These descriptive observations were followed by focused observations based on emergent leads.

### Analysis

Coding following Grounded theory method [21, 22] was begun as soon as data became available, that is, after the first observation. The process of coding started with initial coding, meaning that transcripts were coded line-by-line, this step was performed by CH. Second, focused coding was carried out meaning that the initial codes were synthesized to give meaning to larger pieces of data, this step was carried out by all authors. Third, theoretical coding was performed to link categories and to specify possible relationships between them. Theoretical coding was discussed among all authors until consensus was reached, see Table 2 for an overview of extracts from the coding scheme.

**Table 2:** Coding scheme

*Actor*	Patients	GPs	Patients	GPs	Patients	GPs	Patients	GPs
***Example of codes***	Having trouble swallowing Feeling like crap Being worried	Asking opening questions Asking about “alarm symptoms” Asking to become sure	Showing with hands Touching body	Nodding Gazing Taking notes Summarizing Giving patient time to speak	Asking for confirmation Asking how to facilitate Following instructions	Giving affirmation Acknowledging efforts Commending patient	Talking about what she wants Wanting to rule out certain things	Giving other suggestions Following guidelines Motivating choices
***Sub-categories***	Presenting sensations & emotions	Asking questions to get the picture	Convincing by showing symptoms	Proving that listening	Facilitating examinations Looking for confirmation	Confirming patient Confirming symptoms	Making demands Initiating actions Questioning	Keeping the process on track (In) validating Motivating choices
***Categories***	Justifying care-seeking		Transmitting credibility		Seeking and giving recognition		Balancing expectations with needs	
***Core category***	Negotiating bodily sensations to legitimize access							

## Results

**Fig. 1.**
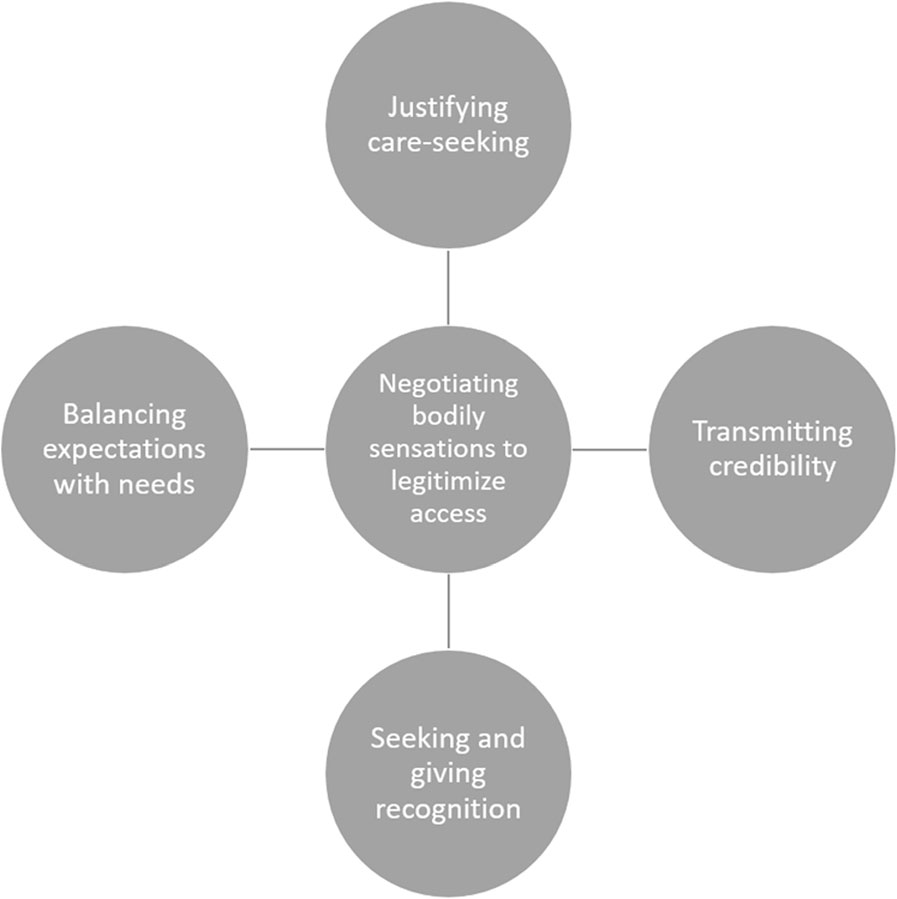
Conceptual model

The conceptual model, developed through the analysis consists of four categories that together build up to one core category, *Negotiating bodily sensations to legitimize access* (See Fig. 1). We interpret the four categories as social processes constructed by the patient and GP interactively within which each employed different strategies of negotiating. These strategies, as we interpret them, were created in response to the actor’s interpretations of the other’s gestures during the encounter rather than as the result of their conscious planning prior to the encounter. These on-going parallel processes during the encounter involved how care-seeking was justified, how credibility was transmitted, how actions were recognized and how expectations were balanced. Combined, these four processes worked to legitimize access to care by negotiating bodily sensations.

### Justifying care-seeking

This category depicted the process of negotiating patients’ reasons for seeking care. In this process, patients used different strategies to convey their health problem while GPs employed different strategies to understand patient’s health problem. This process highlighted how GPs were to a great extent dependent on the patients’ presentations to be able to negotiate the justification of their care-seeking.

Patients’ strategies consisted of using sensations and emotions to convey their reasons for care-seeking. When describing sensations, patients used their emotions to enhance the relevance of their visit to the PHC. That is, they typically followed up their depiction of sensations with argumentation based on emotions. Patients carefully described feelings and emotions that the embodied sensations generated in, for example anxieties and worries.

*‘The patient expresses that she is worried since she have had diarrhea and she thinks it might be a sign of something bad.’ (Observation 3).*

Patients sought care for their interpretations of alarming changes in their body and GPs’ task was to assess these presented sensations. GPs’ strategies consisted of asking questions and gathering relevant information on the experienced sensations to be able to understand patients’ reasons for care-seeking. By using questions, GPs assessed whether the experienced sensations were alarming, a potential serious disease or not and when it was relevant they match them to the standardized CPP template for further fast-track to care, as in this example:

*‘The GP asks “Have you found blood on the toilet paper?” “Yes” the patient responds and explains that this is one reason for her care-seeking.’ (Observation 3).*

Patients’ argumentation often initiated further questions from the GPs who tried to become informed about patients’ sensations and problems, trying to create a picture, in order to assess patients’ problem in accordance to their medical expertise. When patients put forth their sensations and problems they were often guided by the GPs, who asked several questions to get more details about patients’ reasons for seeking care, thereby guiding and steering the encounter. These questions pushed patients to respond and provide information the GPs requested as ways to understand and accurately assess the experienced health complaints, its importance, the need and the appropriate level of care corresponding to these.

*‘The GP steps into the waiting room and calls out the patient’s name, and then show us to her room. The patient sits down in one of the chairs next to the desk, the GP sits down almost opposite to the patient and says “Well, stomach problem?” the patient responds “Yes, that is the thing”. The GP then asks for how long the patient have experienced this problem. (Observation 11).*

During encounters, patients presented, responded and argued for their symptoms and needs. While GPs asked, appraised and reappraised, checked and controlled the information, trying to ensure that they had comprehended the presented information accurately in accordance to their medical expertise, asking leading questions, and by that justify the patient’s reasons for seeking care.

*‘The encounter starts with the GP talking about the patient’s medicine and illness history, which he had been informed about by reading the patient’s medical journal. He then asks the patient to tell him why she is here [at the PHC] today. The patient says that she found blood in her stools a couple of days ago. The GP asks if she has experienced that before and how often it has happened since that day.’ (Observation 15).*

### Transmitting credibility

This category depicted the process of negotiating credibility. In this process, both patients and GPs employed different strategies to convey their message and to persuade each other. Patients employed strategies to convince the GPs of the seriousness of their sensations and symptoms. GPs responded to these strategies by devoting time and attention to listening to the patient. This process illuminated how both the patient and GP strived to achieve credibility where patients often visualized their sensations as symptom alarming something serious, and GPs used both verbal expressions and body language as strategies to appear trustworthy.

As a response to patients’ efforts to be taken seriously, GPs attempted to demonstrate that they were paying attention. With verbal expressions patients showed an effort of trying to convince the GPs that they were credible and that the information they gave was accurate. GPs carefully listened to patients’ stories and reasons for seeking care, commonly repeating back what the patient said, or signaling their attention by, for example, saying “mhmm” or using body language, such as nodding, to prove that they were listening.

Patients often described situations in detail, mirroring an authentic picture and strengthening their reasons for their visits to the PHC. This was illuminated in situations where patients acted to ensure that the information she or he gave was truthful and based on their own experiences and observations. For example:

*‘Patient says “The toilet was red, and the paper I used when I wiped myself was red”. Patient explains that she looked two times to make sure that it was really blood that she had found on her toilet paper. She continues by saying that she was the only one home at that time, so the blood in the toilet must have come from her, and that she was certain that the blood was not there yesterday.’ (Observation 4).*

GPs appeared active and focused during the encounters, most often, by taking notes, which may enhance patients’ feeling of being taken seriously. By asking follow-up questions, GPs ensured that they were listening, gathering more details about sensations, and comprehending given information in order to be able to discern whether there was a serious disease needing a referral for fast medical investigation, such as with CPP. Follow up questions could be about the location of the lump that the patients had experienced in her breast, as a way to verify it and assess following expertise and guidelines. The patient then gave a distinct instruction on how to detect the lump, to convince the GP of the lump’s presence, and by that convince the GP that her visit to the PHC needed to be taken seriously.

Furthermore, patients tried to underpin and reinforce their verbal presentation of experienced sensations with body language to visualize their sensations. Most common, patients did not start the encounter with this visualization strategy, often this took place during a later phase of the encounter. GPs reacted to these visualizations by acknowledging the presented sensations and signs, by for example asking more questions or initiating an examination.

*‘The patient tells the GP about her problem with her gall bladder, which she also gets medication for. During this conversation, patient hinge and grabs her stomach. The GP then asks*” *Is it where you are pointing that hurts?” “Yes it is” the patient answers.’ (Observation 3).*

Conversely, when patients sought care for a visible symptom, such as a deviant skin lesion, it seemed to be less discussion and negotiation between the two parties. Meaning that patients engaged less in presenting their reasons for care-seeking and that GPs did not ask as many questions. In cases where symptoms were easy to verify by direct observation, the GP immediately focused on the lesion itself. Easy visual and objective clinical findings, such as skin lesions, appeared sufficient to be considered, and thereby legitimized, and recognized as a serious alarming symptom needing referral without additional questions and explanations. As soon as the GP stepped into the room and put a glance at the patient’s skin lesion it was verified. This type of symptom approval was not observed when patients seeking care for other alarm symptoms.

*‘The GP steps into the examination room and places himself in front of the patient, looks at the skin lesion and says, within a couple of seconds, “Yeees, well look at that”, and asks the patient how long he have had this skin lesion.’ (Observation 7).*

### Seeking and giving recognition

This category depicted the processing of negotiating the seeking and providing of recognition. It seemed important for patients to present themselves as persons who did “the right thing” and who sought to make it as easy as possible for the GP. They sought GPs’ reactions in the form of acceptance and confirmation of their presented sensations and symptoms, while GPs showed attention to the situation. This process illuminated patients’ need to be acknowledged as patients who had done the best they could to facilitate examination, and GPs’ responses to patients’ desires for confirmation. The process further illuminated patients’ efforts to be understood and accepted in such vulnerable situations when they felt that they had failed with facilitating the examination, or in any other way made it troublesome for the GP.

Patients appeared to want to make sure that they had followed the instructions given before the encounter and get confirmation for doing what they had been told to do. For instance, patients who had been asked to perform an enema beforehand wanted confirmation from the GP that they had successfully accomplished this task. GPs confirmed verbally and gently, which appeared to help patients become relaxed, feel good about themselves, and to make them feel that they had done what was expected from them. During specific examinations, GPs verbally explained what they were going to do, for example:

*During a rectoscopy examination: ‘A couple of minutes passes, it is quiet. Then the GP asks the patient “Is it going well?” patient answers “yes”. A while later, the GP starts a buzzing machine, and stares into the rectoscopy. He then explains that he is going to suck out some fluid and that is the thing which causes the buzzing sound. Patient then asks “Have I managed to empty my bowel?” the GP responds by saying it looks good. The patient appears relived and says “Well that was nice”.’ (Observation 1).*

In several cases, patients explicitly showed that they did not want to cause any trouble or inconvenience for GPs and expressed that they tried to ease the examination. For example, patients asked if they in some way could ease the examination, such as adjusting their position, in order to help doe the GP to perform it maximally well, and facilitate identification of symptoms or disease. However, in cases where patients perceived that they failed with their task of being a compliant patient or in any other way made it challenging for the GPs to perform their job, patients apologized for the inconvenience it might cause. For example, if patients had not succeeded with the enema, was sweaty or failed with following instructions for urine sample.

*‘The patient explains that she, during the night, found blood in her urine when she urinated, which she also found this morning. The GP asks if the blood was flowing or gore, the patients explains that it was gore blood that she found. She also says that she has left a urine sample, but that she was unable to wait 4 hours to urinate for the sample as she had been told to do. The GP confirms that he understands and says that it is okay anyway.’ (Observation 4).*

### Balancing expectations with needs

This category depicted the process of negotiating expectations of the encounter and reach agreement regarding the next step of care. In this process, patients and GPs used different strategies to fulfil, what we perceive as, their own expectations and preferences, and thereby make the encounter successful for both parties. Patients employed strategies such as demanding, initiating actions and questioning, due to sensations experienced as alarming serious illness, while GPs used strategies to either validate or invalidate patients’ demands to keep the process on track and when relevant identify valid reasons for referral for fast investigation by standardized CPP. This process illuminated how GPs, as medical experts, negotiated preferences by motivating their decisions and choices.

Based on their expectations and needs, patients acted and spoke up to become informed, get examined or to get treatment. GPs either validated or invalidated these wishes by accepting or refusing these, based on their medical expertise and the standardized template of CPPs.

*‘The patient says “I want to rule out certain things, It feels good to do what I can”, the GP replies “Well, we have to see how it goes and how we will do”. The patient then asks “But what test will you do, and what can those tell?” The GP explains that he plans to take both electrolytes, calcium and blood status as well as thyroid to rule out any inflammation. The patient appears satisfied and says “Yes, I had something like that in mind”.’ (Observation 9).*

A challenge for GPs appear to be to match patients’ expectations based on their experienced alarming symptoms with GP’s medical expertise. In order to balance the patients’ expressed wishes, suggestions and expectations, GPs argued and motivated their decisions to clarify to patients their reasoning behind these suggestions, trying to bridge their expectations. However, even though GPs motivated their choices, patients did not always automatically show satisfaction and instead continued to argue for their expectations. Therefore, an ongoing polarized discussion sometimes persisted between patients and GPs, putting additional demands on the negotiation between them. When patients appeared dissatisfied with GPs’ suggestions, they opposed, requested and asked for the things they wanted, for example:

*‘The GP says again that she want her [the patient] to leave a stool sample, the patient asks “no blood sample?”, the GP responds and says that she do not think that is necessary. The patient questions this decision, she arguing for a blood sample by saying that it was 1.5 years ago since she had her blood tested. The GP accepts the patient’s request, she says “Well okey then, shall we take a blood status and a CRP [indicator for inflammation and infections]?” The patient agrees and appears satisfied with this decision.’ (Observation 11).*

In addition, patients requested clarity from the GPs and challenged GPs to explain and speak straight and clear, without hiding any information from them. For example, it could be stressed by patients that they wanted GPs to use the word cancer if needed when communicating information to patients, for example:

*After examination: ‘The GP gets up from his desk, walks to his bookshelf and brings out a paper form. He returns to his desk and fills in the form and then says “Since this is something new and I can feel the lump, we will send you for further examination.” Patient replies and expresses that this is difficult but that she also thinks that further examination is necessary. The GP brings forward an informative letter [about CPP], he says “You will get an informative letter, well it says here … well … it says that … eehm … well the C-word is written here.” The patient then interrupts “We can talk about it out loud, I have had that thought myself”. The patient understands that it is cancer that the GP refers to.’ (Observation 5).*

However, GPs motivated and explained carefully why, for example, a referral was necessary to make patients understand the seriousness of their health situation and what was going to happen next and why. GPs motivated their decision by for example referring to and explaining about CPP, when further care was needed. Lastly, GPs typically ended encounters by asking patients whether they had any questions, opening up for dialogue. When patients appeared to doubt GPs’ explanations, assessment and decisions in terms of diagnosis and further treatment, patients often asked “controlling questions” to reassure that their GP had made the right assessment. For example:

*‘The GP explains that it is difficult to know for sure, he repeats the explanation and says one more time that it can be due to scratches in the mucous. Patient then asks “If one would have colon cancer, would you sense it? GP answers by explaining that it can be displayed in different ways.’ (Observation 3).*

## Discussion

Our study demonstrated how bodily sensations were negotiated through four social processes created by patients and GPs and that combined to legitimize further access to care. Negotiation during care-seeking encounters has previously been conceptualized as interactive complex processes affected by participating parties [14, 24], where access to further care and treatment is influenced by context and subjects [25–27]. Hence, patient-provider interaction may contribute to differences in treatment and management for patients with cancer symptoms [26]. However, we found that patients and GPs are mutually dependent on each other, hence the encounter is reliant on the interaction between the two parties, including their different expertise. Our findings illuminated the challenges of matching expectations of doing as good as possible in accordance to frameworks of guidance and expertise. To seek and to give care seemed to be more complex than expected, indicating that the role as patient and the role as GP is more than just a person seeking care vs. a person giving care. The two parties are involved in an asymmetric power relation, yet they are mutually dependent on each other, and the negotiation during encounters encompasses four social processes described in this study.

The first social process we found in negotiation during encounters was justification of patients’ health complaints and symptoms. Merely presenting worries was not sufficient for legitimization to access to further care. Patients were required to argue and operationalize their care-seeking, meaning to be capable to negotiate reasons for care-seeking by justifying embodied sensations with the GP. This finding is significant because previous research regarding how patients present their sensations is scarce. Indeed, most research about patient-provider communication has solely focused on the provider perspective, for instance, GPs’ communication skills [24, 28, 29]. In our study, GPs were challenged to be attentive to patients’ presentations of complaints and needs. Therefore, they asked questions to get as much relevant information as possible, which was needed for assessment of patients’ problem and reconciliation of their own interpretation of the perceived problem with the patients’ experience. However, this illuminates a risk for those who cannot argue for their need of care. Sometimes GPs had to ask specific questions in order to capture the presence of specific alarm symptoms typical for some cancer types, which is in concordance with other studies. In addition, Rogers et al. [30] also found that If such engagement and searching for specific information when communicating with patients is lacking among GPs in primary then there will be a risk of not good and complete comprehension of patients’ illness experiences followed by inappropriate referral and insufficient care.

As mentioned previously, and in line with our results, assessing patients’ presentation of experienced symptoms of cancer is challenging for GPs, since many alarm symptoms are ubiquitous of other diseases, diverse, and often connected with “normal” bodily sensations, hence, diagnosing cancer is complex [1, 4, 31]. The complexity of diagnosing cancer challenges the interaction between patient and GP, demonstrating the mutually dependence of the two involved parties. GPs in our study were dependent on the patient’s presentation of experienced sensations and symptoms and were sometimes required to ask specific questions in the search for specific symptoms. While patients were dependent on the GPs to have their reasons for care-seeking justified, hence being given the possibility to access further care.

The second social process we found in negotiations during encounters was transmitting credibility. Our results indicated that patients perceived a need to make themselves credible, by, for example, visualizing their sensation or in other ways emphasizing the accuracy and the experienced seriousness of their sensations. In addition, GPs in our study used both body language and verbal expressions for appearing as credible medical experts. That patients worked and strived for being perceived as credible was a key finding, also reported in previous research regarding patients’ need of being perceived as reliable when seeking care for symptoms which are conceptualized as medically unexplained [32].

We observed that when patients sought care for symptoms that could be easily verified by eye gaze, less efforts were put into appearing credible. In these cases, GPs were perceived to put less attention towards patients verbal presentations. Interestingly, the phenomenon of hierarchy between bodily sensations and objective medical signs of illness, highlighted in previous literature Risør [8] illuminates the gap between the individual patient and the GP, which may have an impact on the perception of patients’ credibility. Furthermore, the counterbalance between vague sensations (i.e. symptoms typical for cancer) and what, from a medical perspective counts as signs of illness, can generate in that GPs put most attention to the most prominent complain during encounters [18]. The strong focus on the presence of specific symptoms, such as alarm symptoms which are valid and legitimized as indicating disease, may result in that symptoms which are medically unexplained become devaluated [33]. Instead, healthcare needs to be more sensitive to the complexity of cancer diagnosis [18], hence, be more attentive to patients’ presentations of bodily sensations [33] which may indicate cancer. The observed hierarchy of symptoms problematizes the encounter. Patients with symptoms interpreted by GPs as non-alarming or ubiquitous risk being over seen and not prioritized, indicating the challenge of the interaction during encounters.

The third social process we found in negotiations during encounters was giving and receiving recognition. We have described how patients strived for facilitating the encounter, by for example make it as easy as possible for the GP to perform examination, which is also reported in a study exploring the means of being “a good patient”, for adolescents diagnosed with cancer [34]. Weaver et al. concludes that strategies as cooperation, minimizing burden and to ease the healthcare professionals’ task are important features for be perceived as a patient doing good [34]. The fear of wasting doctors’ time have been found among cancer patients, especially among those experiencing vague symptoms [35, 36], and among other patient groups [37]. These findings problematize care-seeking, illuminating patients’ possible concerns of burdening the healthcare organization if they not perceive their sensations and symptoms as legitimated for seeking-care. Our results showed that patients wanted confirmation from their GP during the encounter. On the other hand, Derksen et al. [38] report in their interview study that GPs sometimes perceive that protocol driven care is a barrier for being empathic towards patients during encounters. Hence, adherence to guidelines such as CPPs might influence GPs provision of confirmation. In sum, this third social process illuminated mutually dependence in terms of seeking and providing recognition during the encounter. Patients are dependent on GPs recognition to facilitate the encounter and to feel that their contribution to the encounter is acknowledged. If GPs acknowledge patients’ contributions to the encounter, it could potentially influence patients’ perceptions of being recognized and confirmed when seeking care.

The fourth social process we found in negotiations during encounters was balancing expectations. Patients did often advocate for their wants and needs, while GPs responded by either validating or invalidating them. Patients and GPs in our study did not always have joint expectations and solutions, which caused a polarized discussion with additional argumentations and demands between them. Andersen, Tørring and Vedsted [18] find that such conflicting situations emerge if vague and/or diffuse symptoms are neglected. Our study illuminated the challenges with negotiating sensations during encounters and highlighted the important and challenging task that GPs have, namely to interpret patients experiences together with their own medical expertise. Mutually dependence seem to be prominent phenomenon during encounters while negotiating sensations, which needs to be acknowledged.

Lastly, to our knowledge, there exists no previous study exploring how access is shaped and created during encounters in primary care for patients with cancer symptoms, hence, this study contributes with important knowledge. However, our results evoke further questions regarding patients’ and GPs’ perception of the encounter in this context and in relation to CPPs, since we assume that there exist more important aspects than those observed and reported in this study. The implementation of CPPs results in new routines for GPs that shapes the extent of their role as gatekeepers. Harris with colleagues suggests that factors such as funding, access to special examinations, workload, clinical guidelines, and relationship with colleagues in secondary care are all influencing GPs referral decisions in patients with a potential cancer disease [39]. Our results visualized the challenges with standardized routines and guidelines such as CPPs, since we observed that matching patients experienced sensations with symptoms reported in standardized forms are not always an easy task. Hence, CPPs seem to influence gatekeeping in primary care and potentially challenges the level of person-centred care which could be problematic, since, according to scholars, person-centeredness [40], communication and dialogue are key factors for quality of interaction [10, 11].

### Limitations

Our study has some limitations. Even though primary care encounters are the main gateway to further care, patients attending encounters have already presented sensations that has been legitimized by a district nurse, meaning they have been given an appointment to meet with a GP, hence, being welcomed into primary care. This can be seen as a limitation, therefore, it would be of great interest to study patients’ presentation of sensations during their first contact with healthcare, which is the contact when calling primary healthcare, or using web-based platform (1177.se).

Additionally, when conducting observational studies, there is always a risk that the observed subject is affected by the observers’ presence, in this case patients and GPs. However, Roter and Hall [10] states that patients and GPs, in previous studies, have reported that they forgot about being observed. Furthermore, a study comparing video recordings of encounters with GPs who knew that they were recorded with GPs who was uninformed found no significant differences in neither length of the encounter nor the numbers of health issues discussed during the encounter [41]. We believe that these findings are applicable to our study, also, since the researchers numerous times informed about the aim of the study, we assess that the risk of observed subject moderate their behaviors is limited. Additionally, observations as a method for data collection have been found to be informative and a cost-effective, especially when studying, for example, quality of care, processes and communication. Observations enable understandings of complex care environments and has been stated to be a valuable method for enabling understandings of complex and dynamic situations [42], such as interactions during encounters. Also, all communication, verbal and non-verbal is a dynamic process, by using observations as a method for data collection it is possible to gain awareness of delicate behaviors and these dynamic processes [42].

## Conclusion

We have shown that the encounter is a complex process, encompassing several social processes were negotiation is key. We assume that it might be more challenging than expected to be adherent to standardized routines and at the same time employ person-centered care and take patients wants into account. Patients and GPs seem to be mutually dependent on each other and both patients’ expertise and GPs’ medical expertise need to be reconciled during the encounter. The four social processes reported in this study acknowledge the challenging task which both patients and primary healthcare face. Namely, negotiating sensations signaling possible cancer and further identifying and matching them with the best pathway for investigations, corresponding as well to patients’ needs as to standardized routines as CPPs. Based on our results, we advocate for a vigilant discussion about how the healthcare organizations can decrease patients’ experienced need of justifying their care seeking. Also, a confirmative approach towards patients as well as a clear and straight communication would most likely be of help.

We recommend further research in other regions, including metropolitan areas and private PHCs. We also suggest that future studies adopt and test our model from this study. It would be of interest to use the model in focus group discussions with healthcare professionals, as well as with medical students, to explore if the model is applicable and useful in praxis. It would also be of interest to explore patients’ perception of these encounters.

## Supplementary information

**Additional file 1.** COREQ (COnsolidated criteria for REporting Qualitative research) Checklist.

## Data Availability

The data that support the findings of this study are not publicly available since the data consists of information that could compromise research participants’ privacy and consent.

## Abbreviations

CPPs: Standardized Cancer Patient Pathways
GP: General practitioner
GTM: Grounded Theory Method
PHC: Primary Healthcare Center
SI: Symbolic Interactionism
